# Diacetyl strategy for synthesis of NHAc containing glycans: enhancing glycosylation reactivity via diacetyl imide protection

**DOI:** 10.3389/fchem.2023.1319883

**Published:** 2023-12-05

**Authors:** Koichi Fukase, Yoshiyuki Manabe, Atsushi Shimoyama

**Affiliations:** ^1^ Department of Chemistry, Graduate School of Science, Osaka University, Osaka, Japan; ^2^ Forefront Research Center, Graduate School of Science, Osaka University, Osaka, Japan

**Keywords:** glycosylation, amide bond, amide, oligosaccharide, glycan, flow reaction

## Abstract

The presence of NHAc groups in the substrates (both glycosyl donors and acceptors) significantly reduced the reactivity of glycosylation. This decrease was attributed to the NHAc groups forming intermolecular hydrogen bonds by the NHAc groups, thereby reducing molecular mobility. Hence, a diacetyl strategy involving the temporary conversion of NHAc to diacetyl imide (NAc_2_) was developed for the synthesis of NHAc-containing glycans. This strategy has two significant advantages for oligosaccharide synthesis. The NAc_2_ protection of NHAc substantially enhances the rate of glycosylation reactions, resulting in improved yields. Moreover, NAc_2_ can be readily reverted to NHAc by the simple removal of one acetyl group under mild basic conditions, obviating the necessity for treating the polar amino group. We have achieved the efficient synthesis of oligosaccharides containing GlcNHAc and *N*-glycans containing sialic acid using the diacetyl strategy.

## 1 Introduction

Natural glycans play diverse roles in biological events such as self and non-self-recognition, viral and bacterial infection, immunoregulation, cancer invasion, and cell development (Zhao et al., 2008; Macauley et al., 2014; Raman et al., 2016; Ballal and Inamdar, 2018; Shirakawa et al., 2021; Manabe and Fukase, 2023). Bacterial glycoconjugates, including lipopolysaccharide and peptidoglycan, have been known to activate innate immunity (Kusumoto et al., 2010; Shimoyama and Fukase, 2023). Owing to the high diversity and heterogeneity of natural glycans, the production of pure glycans through chemical synthesis has proven to be a potent method for understanding the biological functions of glycans.

Amino sugars are essential components of various glycans including *N*-glycans, *O*-glycans, glycolipids, glycosaminoglycans, and bacterial cell walls. Glucosamine, galactosamine, and sialic acids are common amino sugars that usually exist in *N*-acylated or *N*-sulfated forms. The choice of protecting groups for amines is crucial in synthesizing glycans containing amino sugars because it affects glycosylation reactivity. Various protecting groups, such as 2,2,2-trichloroethoxycarbonyl (Kusumoto et al., 1985; Imoto et al., 1987), allyloxycarbonyl (Boullanger et al., 1987; Boullanger et al., 1990), trichloroacetyl (Wolfrom and Bhat, 1967; Blatter et al., 1994), trifluoroacetyl (Meyer zu Reckendorf and Wassiliadou-Micheli, 1970), and phthaloyl (Lemieux et al., 1977) have been reported to protect amino sugars. The azide group is also a useful precursor of the amino group (Lemieux and Ratcliffe, 1979). Since most amino groups are acetylated in natural glycans, protected amino groups must be converted to acetamide (NHAc) through acetylation. These steps can be avoided when glycans with NHAc are used directly to construct glycan chains. However, fragments containing NHAc exhibit low reactivity in glycosylation although Tamura et al. showed that the formation of interglycosidic *O*-imidates with NHAc in GalNAc increases glycosylation yields (Tamura et al., 2008). Boons et al. demonstrated that NAc_2_ sialyl donors exhibit significantly higher reactivity than NHAc donors (Demchenko and Boons, 1998). Kononov et al. observed hydrogen bonding of NHAc in Neu5Ac donors, and reactivity improvement was achieved by protecting NHAc as NAc_2_ or adding external amides/imides to disrupt the hydrogen bond (Kononov et al., 2008; Kononov et al., 2009; Kononov et al., 2012). Crich et al. reported that intermolecular hydrogen bonding formed by NHAc in GlcNAc acceptors reduces reactivity (Crich and Dudkin, 2001). Auzanneau observed the aggregation of a tetrasaccharide through hydrogen bonding of NHAc in GlcNAc using NMR (Kuir et al., 2015).

We also observed that sialic acid and GlcNAc derivatives containing NHAc or fragments that include them displayed low reactivity (Nagasaki et al., 2016; Zhou et al., 2016; Tsutsui et al., 2020). This could be attributed to the formation of intermolecular hydrogen bonds between *N*-acetylneuraminic acid and GlcNAc derivatives, which restrict the molecular movement and hinder the access of acceptors to donors, as noted by Kononov and Crich. We therefore developed a “diacetyl strategy” by temporarily converting NHAc to diacetyl imide (NAc_2_). The diacetyl strategy offers two advantages for oligosaccharide synthesis. NAc_2_ protection of NHAc significantly enhanced the glycosylation reactions, leading to improved yields. Additionally, NAc_2_ can be easily converted back to NHAc by removing one acetyl group under mild basic conditions, eliminating the need to treat the polar amino group (Nagasaki et al., 2016; Zhou et al., 2016; Tsutsui et al., 2020; Shirakawa et al., 2021).

## 2 Comparison of reactivity of C5-acetamide sialic acid donor with other sialic acid donors

Much effort has been devoted to the development of efficient and stereoselective α-sialylation. This reaction was one of the most challenging in the field of oligosaccharide synthesis because α-sialylation poses steric and electronic difficulties. Recent advances in glycosylation chemistry such as the development of new leaving and protecting groups have enabled efficient construction of α-sialoside linkages.

As mentioned above, the reactivity of sialic acid derivatives possessing a C5-acetamide group is low. Thus, new sialyl donors with various C5 substituents, including protecting groups and functional groups such as carbamates, have been developed to improve the reactivity and stereoselectivity in α-sialylation. The kinetic solvent effect of nitriles has been used for α-sialylation, except in the case of cyclic carbamates and macrocyclic donors described below. Conducting reactions at lower temperatures generally enhances selectivity in kinetically controlled reactions; thus, the improvement in reactivity contributes to the enhancement of stereoselectivity. These substituents include *N*-Ac_2_ (Demchenko and Boons, 1998), *N*-TFA (Meo et al., 2001; Ando et al., 2005), *N*-Troc (Ando et al., 2005; 2003; Adachi et al., 2004), *N*-phthaloyl (Tanaka K. et al., 2005), azide (Yu et al., 2001), isothiocyanato (Mandhapati et al., 2015), and ureido groups (Tanase et al., 2016). 5,4-*N*,*O*-cyclic carbamates have been utilized for highly selective α-sialylation, especially in the α (2–8)- and α (2–9)-sialylation cases (Tanaka H. et al., 2006; 2008; 2009; De Meo et al., 2008; Hanashima et al., 2009; Chu et al., 2011). A highly selective sialylation method was developed using a macrocyclic α-sialyl donor bridged by alkyl chains at the C1 and C5 positions of sialic acids (Komura et al., 2019; Vibhute et al., 2021). In this method, nucleophilic attack is possible only from the α-face because of the blocking of the β-face by the tethered moiety.

## 3 Glycosylation under microflow conditions: enhanced yield and selectivity through improved mixing efficiency and precise temperature control

Microflow synthesis is characterized by its ability to remove heat rapidly and efficiently, maintain precise temperature control, and facilitate effective mixing. Consequently, microflow synthesis has been employed to enhance the reaction rate and improve yields in a biphasic system involving water and organic solvents, primarily due to its efficient mixing capabilities (Liu et al., 2004; Tanaka K. et al., 2008; Otake et al., 2018; Aimi, 2021).

On the other hand, glycosylation reactions are conducted in a homogeneous reaction system in organic solvents under dry conditions. However, as pointed out by Kononov et al., protected sugar derivatives may not be uniformly dispersed in solution; instead, they often tend to form clusters because of interactions such as intermolecular hydrogen bonding (Kononov, 2015; Kononov et al., 2017; Podvalnyy et al., 2017; Orlova et al., 2017; Nagornaya et al., 2018; Myachin and Kononov, 2023a; Myachin and Kononov, 2023b). Microflow mixing of glycosyl donor and/or receptor clusters can afford a non-equilibrium mixing state similar to well-dispersed biphasic mixture. Therefore, the use of microreactors, as with biphasic reactions, can influence both the yield and selectivity.

We observed a similar phenomenon in the synthesis of partial structures of *Helicobacter pylori*-derived lipopolysaccharides. Lipopolysaccharides consist of a glycolipid portion called lipid A and a polysaccharide part, with the polysaccharide part being linked to lipid A through an acidic sugar termed Kdo (3-deoxy-D-*manno*-oct-2-ulosonic acid). In the synthesis of Kdo-lipid A, we developed Kdo glycosyl donor **1** protected with isopropylidene at the 4th and 5th hydroxyl groups and containing *N*-phenyltrifluoroacetimidate as a leaving group (Shimoyama et al., 2011a; Shimoyama et al., 2011b). Compound **1** adopts a boat conformation, with the isopropylidene group covering the β-face of Kdo. As a result, in glycosylation using **1**, sugar acceptors attack from the α-direction, leading to a high selectivity for α-glycosides. However, a substantial amount of glycal **4** was generated as a byproduct through intramolecular elimination reactions, and a considerable excess of **1** (5 equivalents) was used to obtain the desired trisaccharide **3** in 70% yield.

**FIGURE 1:**
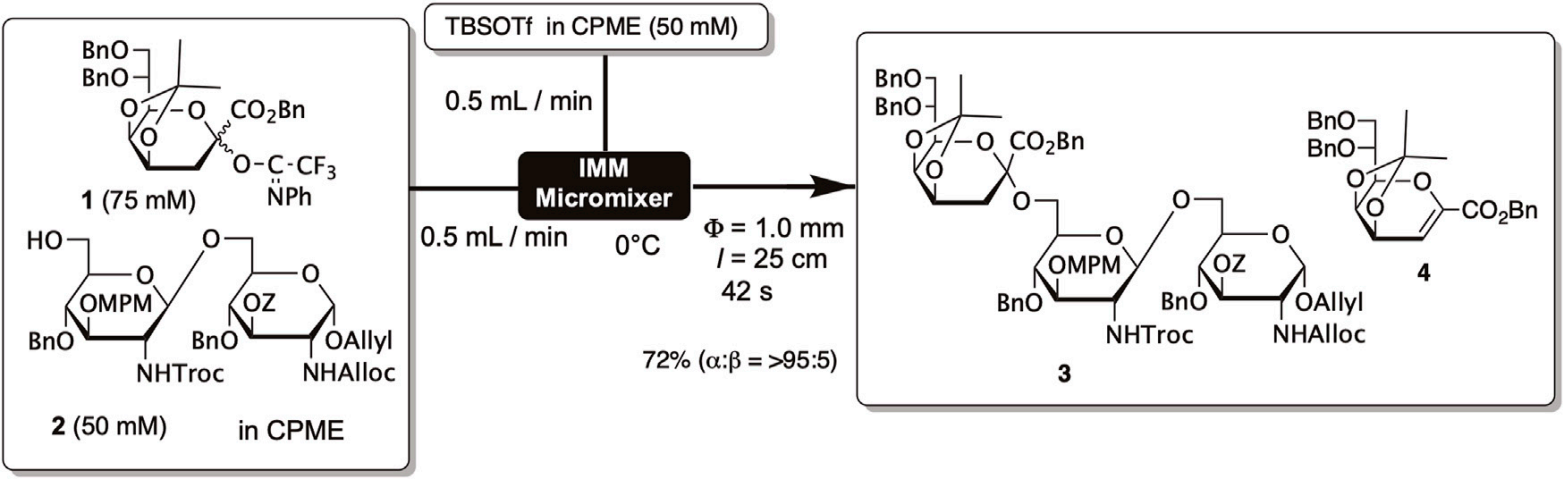
Glycosylation of Kdo donor by using the microreactor.

For the total synthesis of Kdo-lipid A, microflow synthesis was employed to increase the efficiency of glycosylation. The solution of glycosyl donor **1** and glycosyl acceptor **2** in cyclopentyl methyl ether (CPME) and the solution of TBSOTf in CPME were mixed using a micro-mixer, allowing the reaction to proceed for 42 s in a tube reactor (Figure 1). This method enabled us to reduce the amount of glycosyl acceptor to 1.5 equivalents, yet we still obtained trisaccharide **3** with a 72% yield, which is almost equivalent to the batch process. This result suggests that efficient mixing disrupts the solute clusters and promotes intermolecular reactions.

**FIGURE 2:**
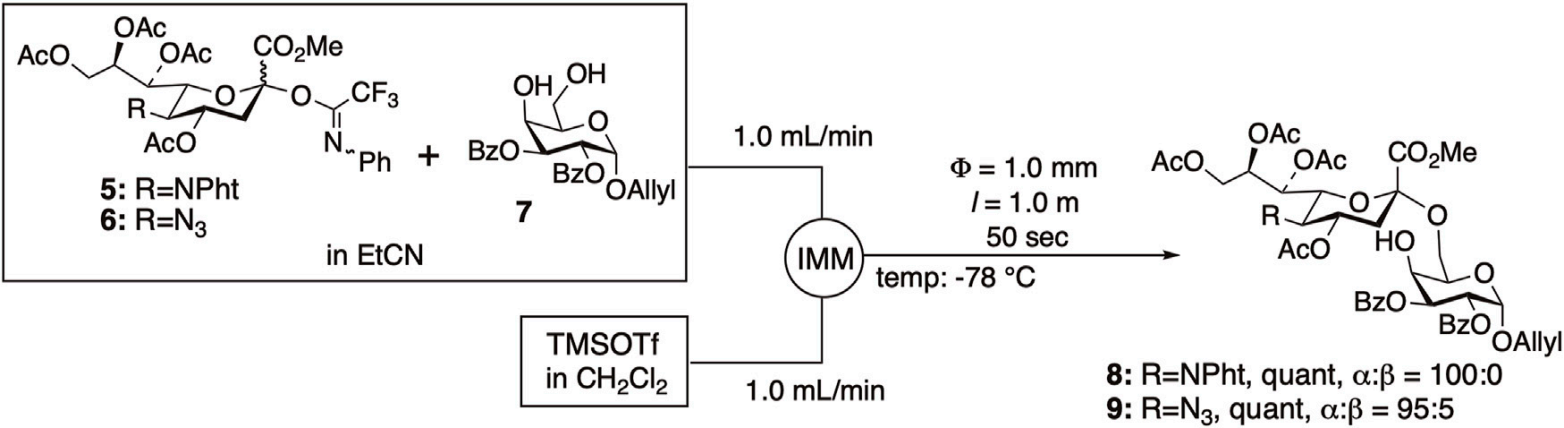
α-Sialylation under microfluidic conditions.

We have developed other microfluidic glycosylation reactions, including α-sialylation (Tanaka K. et al., 2005; Tanaka S et al., 2007; Uchinashi, Y. et al., 2014), β-mannosylation (Takaka, K. et al., 2009), and *N*-glycosylation of asparagine (Takaka, K. et al., 2009), many of which have successfully improved both selectivity and yield. The main factor thought to contribute to the improvement in selectivity and yield is efficient temperature control; however, enhanced mixing efficiency may also play a role in the improvement. We achieved quantitative sialylation using *N*-phenyltrifluoroacetimidate donors **5** and **6** with C5-phthalimide (Tanaka K. et al., 2005; Tanaka S et al., 2007) or azide (Uchinashi, Y. et al., 2014) with high α-selectivity under microfluidic conditions (Figure 2).

**FIGURE 3:**
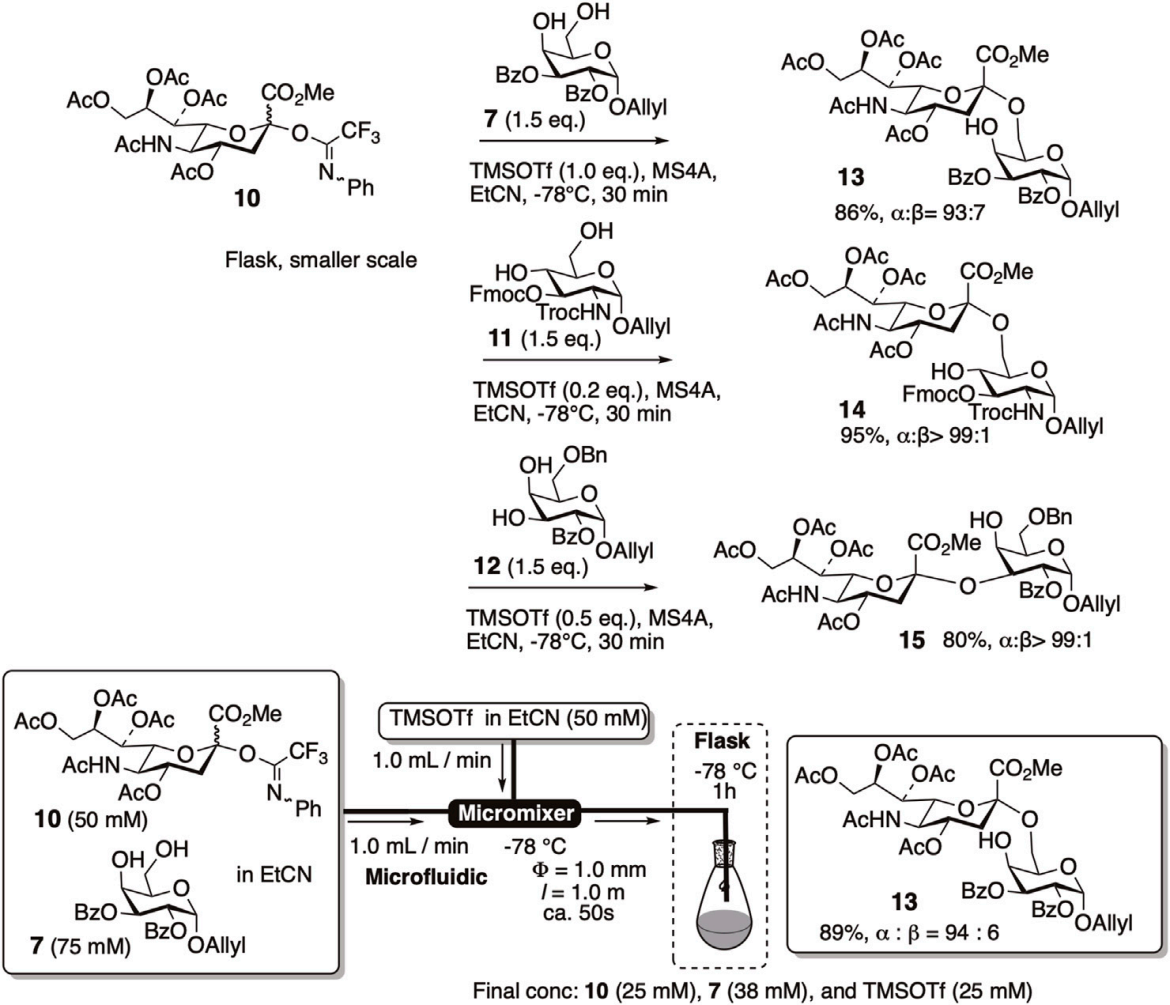
Re-investigation of α-sialylation using C5-acetamide donor **10** under batch and microfluidic conditions.

Despite previous studies describing low to modest efficiency, we re-examined the readily available C5-acetamide donor **10** for its application in α-sialylation under batch and microfluidic conditions (Figure 3). *N*-Phenyltrifluoroacetimidate donor **10** was efficiently mixed with an appropriate amount of TMSOTf to yield α (2–6) and α (2–3)-sialylation products of galactose and glucosamine acceptors with excellent yields and high α-selectivity (Uchinashi, Y. et al., 2011).

## 4 Diacetyl strategy

### 4.1 Diacetyl strategy for the synthesis of GlcNAc containing oligosaccharides

**FIGURE 4:**
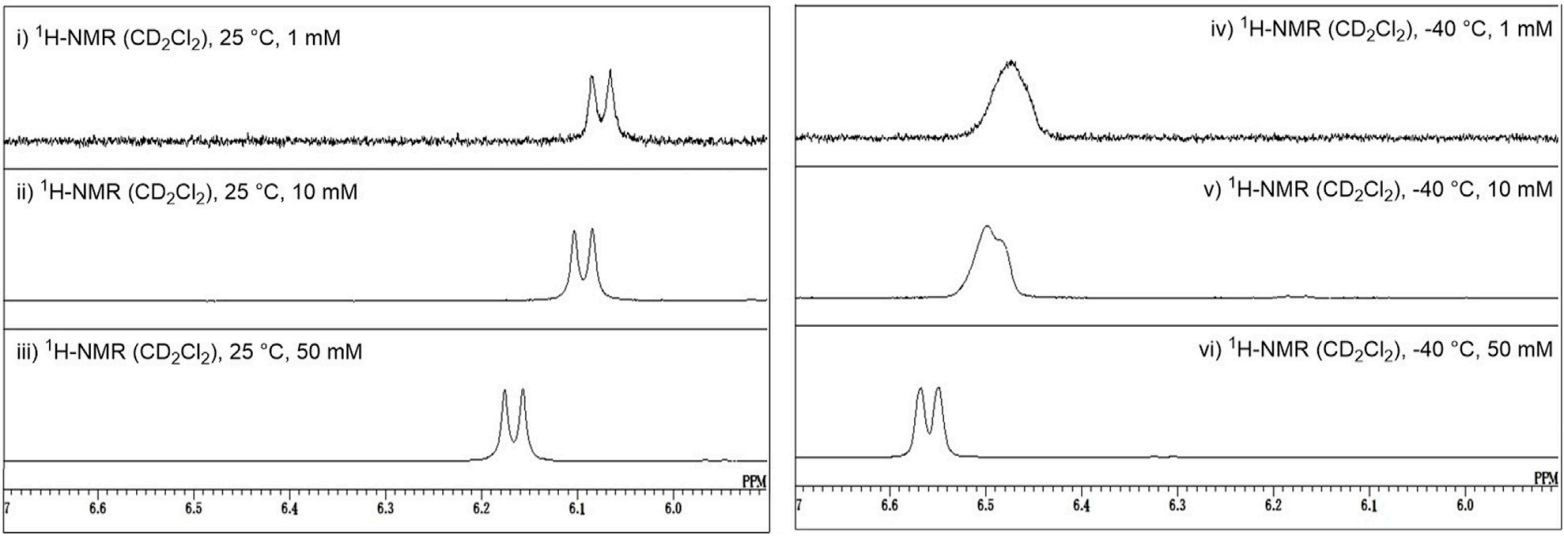
Concentration and temperature dependence of the ^1^H-NMR spectrum of acetamide acceptor **17**, where downfield shifts of the amide proton are observed upon increasing the concentration and decreasing the temperature.

**FIGURE 5:**
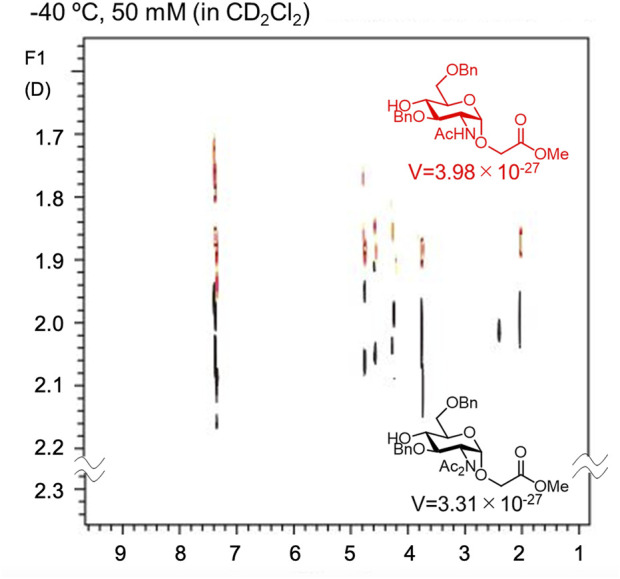
DOSY spectrum of *N*-acetylated (red) and *N,N*-diacetylated (black) glucosamine derivatives. The effective volume (V) was estimated by Stokes–Einstein equation.

As described above, Crich et al. demonstrated that *N*-acetylated glucosamine acceptors exhibit lower reactivity than *N*-phthaolyl glucosamine and 2-azido-2deoxy glucose derivatives. They attributed the reduced reactivity to intermolecular hydrogen bonding, which was confirmed by the concentration-dependent chemical shift of NHAc in ^1^H-NMR. We also confirmed that the NHAc group of GlcNAc derivative **17** formed intermolecular hydrogen bonds based on the concentration- and temperature-dependent chemical shift of the amide proton in the ^1^H-NMR spectrum in CH_2_Cl_2_ (Figure 4) (Tsutsui et al., 2020). The supramolecular structure of GlcNAc **17** formed by intermolecular hydrogen bonds was observed using diffusion-ordered two-dimensional nuclear magnetic resonance spectroscopy (DOSY) measurements (Figure 5). The effective volume of GlcNAc **17** was 1.2 times larger than that of *N,N*-diacetylated glucosamine (GlcN(Ac)_2_) **18** according to the DOSY spectra, indicating that **17** formed a supramolecular structure through intermolecular hydrogen bonds, whereas **18** did not aggregate. This slight difference suggests that the hydrogen bonds formed by NHAc are likely dynamic but still have a significant impact on the reactivity of GlcNAc.

**FIGURE 6:**
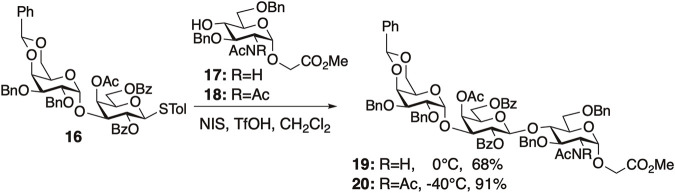
The [2 + 1] glycosylation using *N*-acetylated acceptor **17** or *N,N*-diacetylated acceptor **18** for the synthesis of protected α-gal **19** or **20**.

Next, we demonstrated the effectiveness of the diacetyl strategy in the synthesis of α-gal trisaccharide and H antigen trisaccharide. As anticipated, diacetyl protection significantly enhanced the reactivity of all the tested glycosylations. After the preparation of thiodisaccharide **16**, the glycosylation of glucosaminyl acceptors **17** or **18** with **16** was performed to evaluate the effectiveness of the diacetyl strategy (Figure 6). The reaction of *N,N*-diacetylated acceptor **18** with **16** proceeded more smoothly than that of **17** with **16**, yielding trisaccharide **20** in excellent yield. These results indicated that the formation of hydrogen bonds by NHAc in **17** decreased its reactivity and demonstrated the effectiveness of the diacetyl strategy.

**FIGURE 7:**
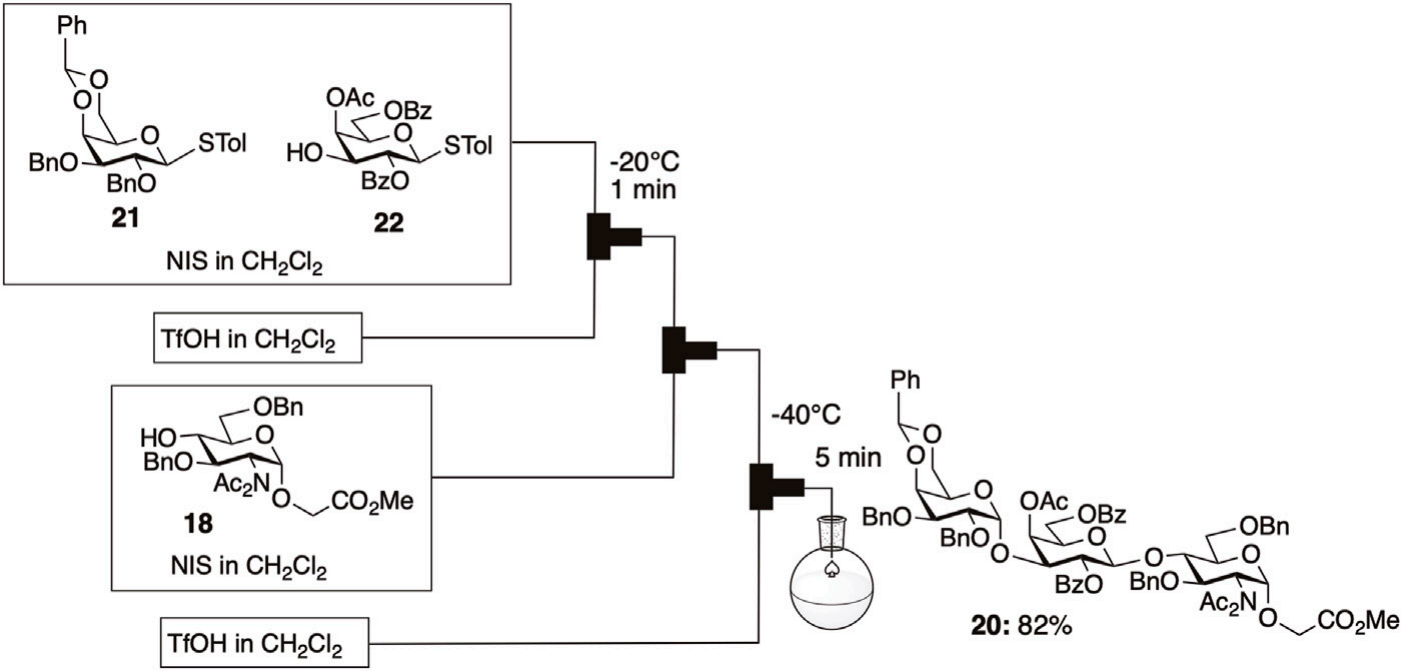
One-flow synthesis of protected α-gal **20** under microfluidic conditions.

Efficient α-gal synthesis was accomplished through one-pot and one-flow glycosylation employing the armed-disarmed strategy, benefiting from enhanced reactivity due to diacetylation. The one-flow method under microflow conditions enhanced the reproducibility of the consecutive glycosylations (Figure 7). This was the first report of one-flow glycosylation utilizing the armed-disarmed strategy, which offers distinct advantages in terms of reproducibility compared to one-pot glycosylation in a batch system.

**FIGURE 8:**
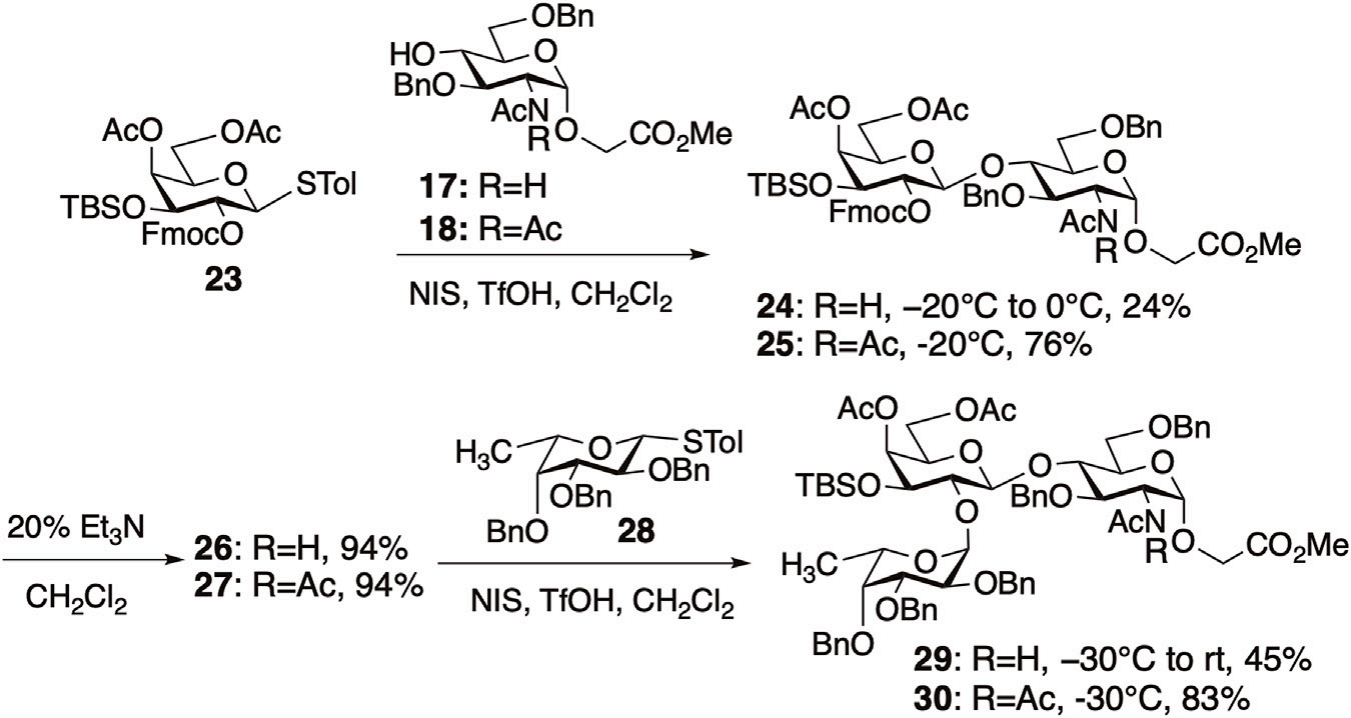
Synthesis of H-antigen trisaccharides.

In H antigen synthesis, enhancement of reactivity by NAc_2_ protection was observed in both glycosylations proximal and distal to GlcNAc (Figure 8). We investigated the glycosylation of GlcNAc **17** and GlcN(Ac)_2_ **18** with a galactosyl donor **23**. As expected, glycosylation with **17** was less reactive, requiring an increase in temperature to 0°C to give compound **24** in 24% yield. In contrast, glycosylation with **18** proceeded smoothly at −20°C, affording disaccharide **25** with perfect β-selectivity in 76% yield.

Next, we compared the reactivity of the disaccharide acceptors **26** (*N*-acetylated) and **27** (*N,N*-diacetylated) after deprotection of the Fmoc groups in **24** and **25**. The glycosylation of **26** with fucosyl donor **28** did not proceed at −30°C; however, when the reaction temperature was elevated to room temperature (rt), trisaccharide **29** was obtained in 45% yield. In contrast, the glycosylation of **27** proceeded smoothly at −30°C to give **30** in 83% yield. This illustrates that *N,N*-diacetyl protection enhances reactivity even when the reactive sites are distant from the NAc_2_ group, underscoring the versatility of the diacetyl strategy in synthesizing glycans containing GlcNAc.

### 4.2 Diacetyl strategy for the synthesis of sialic acid containing oligosaccharides

**FIGURE 9:**
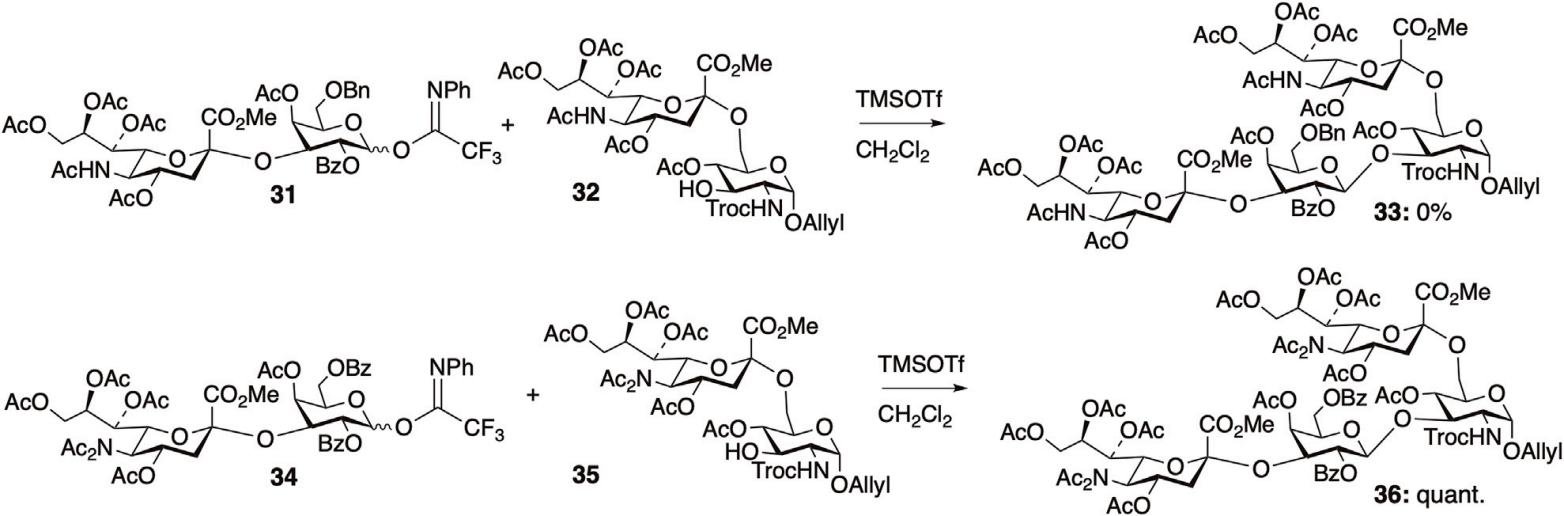
Synthesis of disialylated tetrasaccharide.

We also reported a “diacetyl strategy” for the efficient synthesis of sialyl glycans. Disialylated tetrasaccharide (Neu5Ac (α2,3) Gal (β1,3) [Neu5Ac (α2,6)] GlcNAc) is a structural motif found in the *N*-glycans of human Factor X and fetuin, gangliosides in human colon adenocarcinoma, and human milk. For the synthesis of this motif, we investigated a convergent route based on the glycosylation of two sialylated disaccharides (Figure 9) (Zhou et al., 2016). The reactivity of the glycosylation reactions between two sialyl disaccharides **31** and **32** with NHAc at the C5 position of sialic acid residues was found to be extremely low, resulting in no desired product formation (0% yield). However, NAc_2_ protection of both sialyl fragments **34** and **35** significantly enhanced the reactivity, leading to the quantitative yield of the desired tetrasaccharide **36**. Protection of the amide group of the sialic acid residues resulted in a substantial improvement in the glycosylation yield between the two sialylated disaccharides, suggesting that the presence of hydrogen bonding on the sialic acid residues decreases reactivity.

Asparagine-linked (*N*-linked) glycans in glycoproteins (*N*-glycans) are oligosaccharides present in eukaryotes and some prokaryotes that display a broad spectrum of structural diversity. These can be classified into three categories: high-mannose type, complex type, and hybrid type. Typically, *N*-glycans exhibit heterogeneity even at specific glycosylation sites. Complex *N*-glycans play crucial roles in various biological processes and diseases, including regulation of glycoprotein dynamics, cell development, immunity, and cancer invasion.

We aimed to synthesize sialylated *N*-glycans, including core fucose-containing disialylated biantennary *N*-glycans, disialylated biantennary *N*-glycans with asymmetrically deuterated sialic acid acetyl groups, and fully sialylated tetraantennary *N*-glycans, using a diacetyl strategy, and we successfully achieved their synthesis (Nagasaki et al., 2016; Shirakawa et al., 2021).

The synthesis of *N*-glycans has been extensively investigated by several research groups. Danishefsky (Wu et al., 2006; Wang et al., 2009; Walczak and Danishefsky, 2012; Walczak et al., 2013) and Unverzagt (Schuberth and Unverzagt, 2005; Eller et al., 2007; Mönnich et al., 2016; Luber et al., 2018) successfully chemically synthesized *N*-glycans, including those with core fucose and bisecting GlcNAc. Ito (Koizumi et al., 2013), Boons (Wang et al., 2013; Li et al., 2016; Gagarinov et al., 2017), Wang (Li et al., 2015), and Wong (Shivatare et al., 2013; 2016) constructed *N*-glycan libraries through a combination of chemical and enzymatic synthesis.

Our approach to *N*-glycan synthesis is characterized by two key aspects: 1) the practical synthesis of fragments using micro-flow reactions, as described above, and 2) the development of a convergent synthetic route for the efficient assembly of *N*-glycan skeletons. Another distinctive feature of our *N*-glycan synthesis is the early-stage incorporation of asparagine (Asn). Glycans were extended from the Asn-containing reducing-end fragment, which was obtained through *N*-glycosylation, as described above. This approach facilitates the straightforward preparation of glycoconjugates, including glycopeptides and glycoproteins, as it eliminates the need for Asn attachment following deprotection.

#### 4.2.1 Synthesis of core-fucosylated *N*-glycan by the convergent synthetic route

Mammalian core fucose is added to complex-type glycans by fucosyltransferase 8 (FUT8) via α (1–6) fucosyl linkages. Knockout mice lacking FUT8 exhibit severe developmental delays and a 70% mortality rate within the first 3 days of life. Core-fucosylated immunoglobulin G (IgG) is significantly less functional (100-fold weaker) in antibody-dependent cell-mediated cytotoxicity (ADCC) than non-core-fucosylated IgG. The importance of core-fucosylation is evident in various stages of carcinogenesis and tumor progression. Reduced core-fucosylation in human colon carcinomas leads to resistance against TRAIL-induced apoptosis and immune evasion. In hepatocellular carcinoma, elevated levels of core-fucosylated α-fetoprotein serve as a valuable marker. Additionally, upregulation of core-fucosylation on growth factor receptors, such as epidermal growth factor receptor (EGFR) and fibroblast growth factor receptor (FGFR), has been associated with receptor activation.

**FIGURE 10:**
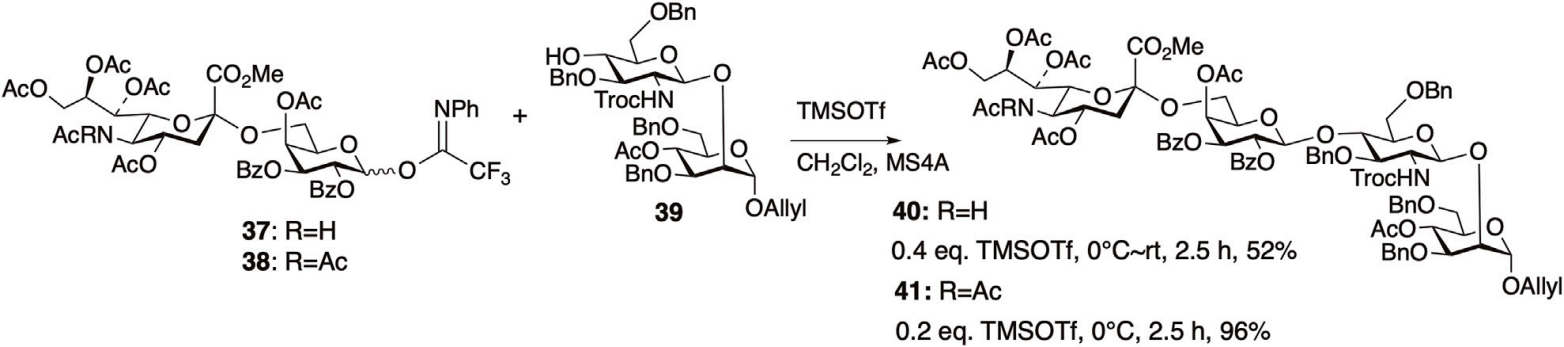
Comparison of reactivity of NHAc donor **37** and NAc_2_ donor **38**.

**FIGURE 11:**
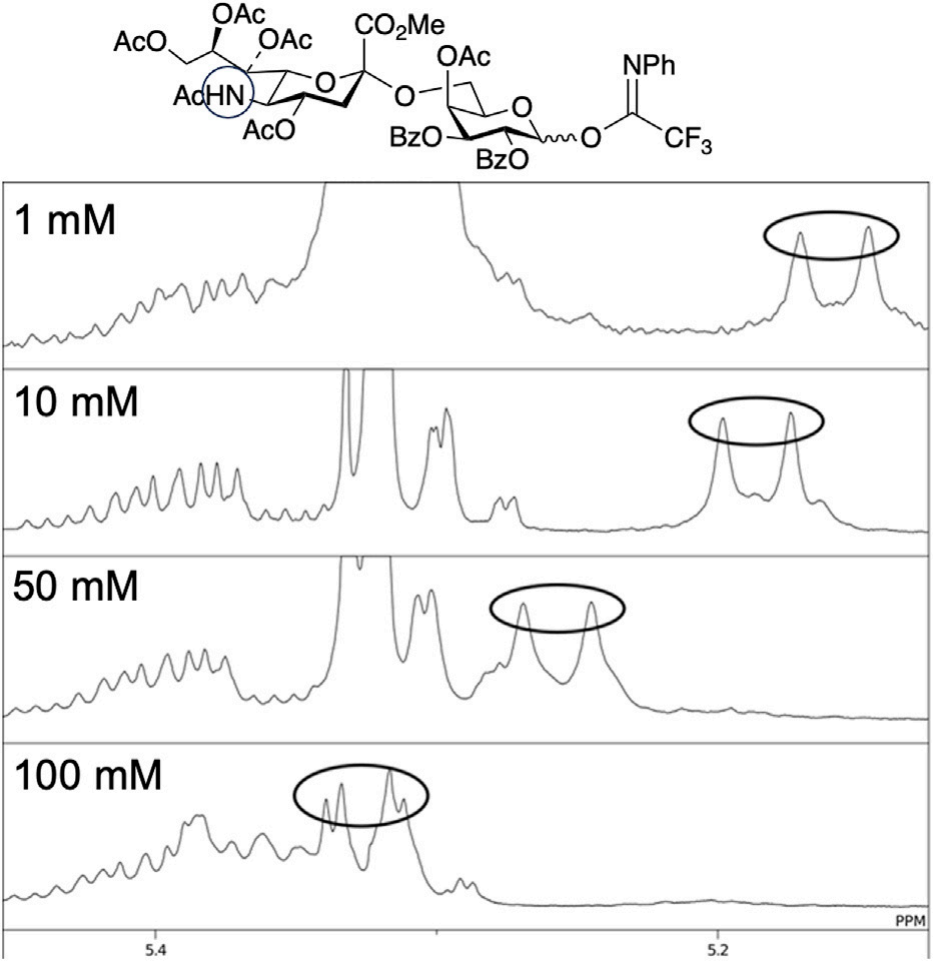
^1^H NMR of the disaccharide donor **37** at various concentrations.

We first examined the synthesis of the non-reducing-end tetrasaccharide in the synthesis of the core-fucosylated *N*-glycan (Nagasaki et al., 2016) (Figure 10). When attempting glycosylation between the NHAc-containing sialyl disaccharide donor **37** and the disaccharide acceptor **39**, only 52% of the desired tetrasaccharide **40** was obtained, even after raising the temperature to room temperature. On the other hand, using **38** where the NHAc at the 5-position of sialic acid was converted to NAc_2_, improved the reactivity. Glycosylation proceeded rapidly at 0°C, yielding the desired tetrasaccharide **41** in 96% yield. We observed a concentration-dependent chemical shift of the amide protons in NMR measurements at various concentrations of **37** (Figure 11), revealing that the NHAc in **37** forms intermolecular hydrogen bonds. This also highlights the significant impact of supramolecular structures formed through hydrogen bonding on reactivity.

Solvent selection played a crucial role in the glycosylation of the reducing-end tetrasaccharide **42** with the non-reducing-end tetrasaccharide **43**. When ether-based solvents were used, particularly cyclopentyl methyl ether (CPME), the desired octasaccharide was obtained in 91% yield. The ether solvents probably stabilize and prolong the lifetime of the intermediate oxocarbenium ion by coordination. However, the stereoselectivity of this glycosylation was moderate (α/β = 3/1). Following cleavage of the benzylidene group from the obtained octasaccharide, the α-isomer **44** was separated. Subsequently, glycosylation at the 6th position of the branched mannose in **44** was investigated. When CPME was used as the solvent, the desired product **45** was obtained in high yield. However, α/β selectivity remained low, and **45** was obtained as a 1/1 mixture.

**FIGURE 12:**
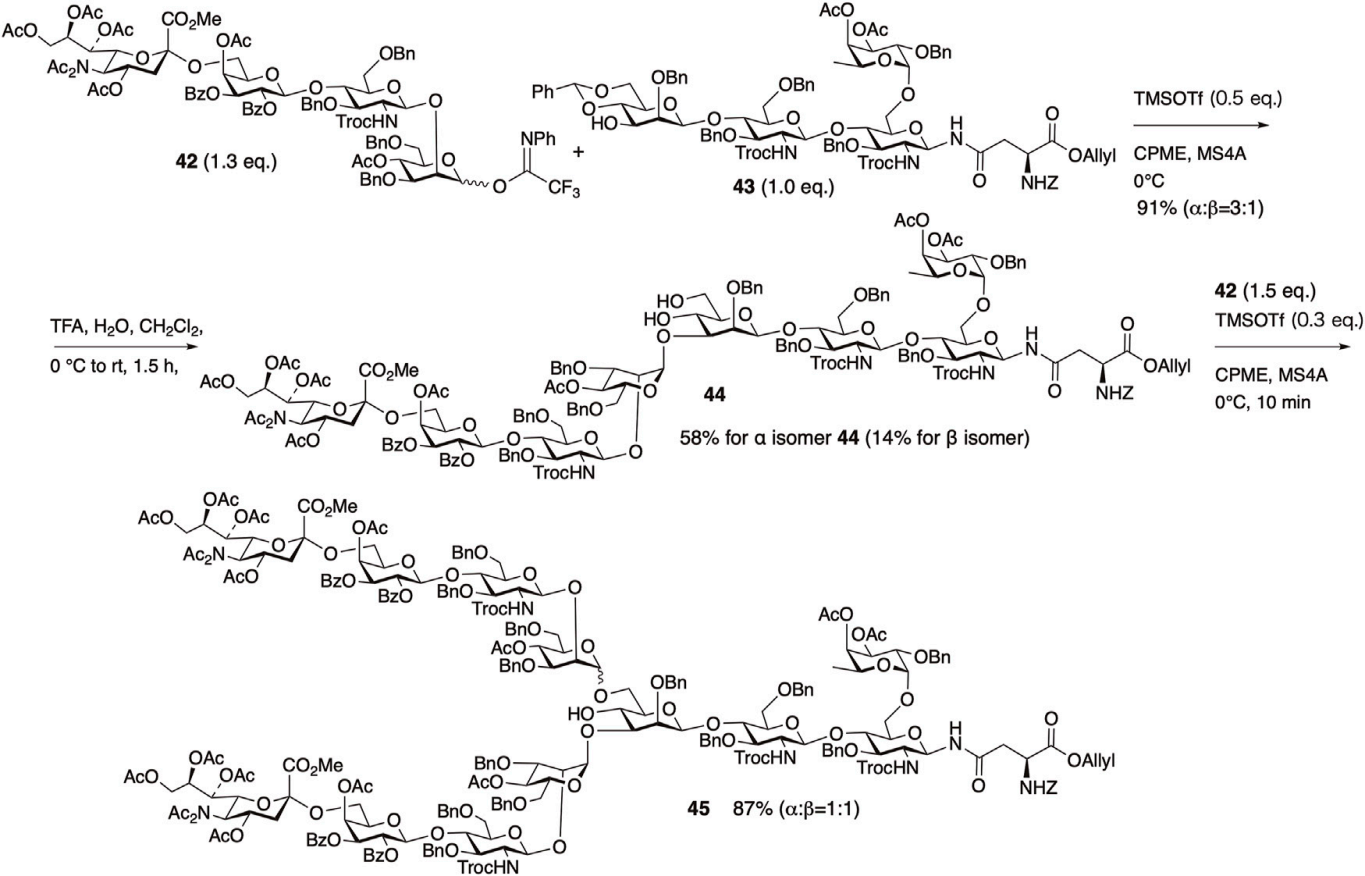
Synthesis of core fucosylated *N*-glycan.

After the cleavage of allyl ester of **45** with Pd(OAc)_2_, PPh_3_, sodium 2-ethylhexanoate in acetone, all acyl groups, *N*-Troc groups, and methyl esters were removed by aqueous LiOH and the resulting amino groups were acetylated. Both the α and *β* isomers were then separated using HPLC, and all benzyl-type protecting groups were removed under catalytic hydrogenation conditions to afford the core fucose-containing *N*-glycan (Figure 12). Protection of the acetamide in sialic acid as a diacetyl moiety may have facilitated the efficient glycosylation of each fragment coupling. The problem of low selectivity in the two glycosylation steps was resolved by remote participation using *O*-3 and *O*-6 acyl protection of the mannose residue in the synthesis of *N*-glycans containing bisecting GlcNAc, along with the synthesis of the previously mentioned deuterated disialylated biantennary *N*-glycans and the fully sialylated tetraantennary *N*-glycan (Manabe et al., 2018; Shirakawa et al., 2021).

#### 4.2.2 Synthesis of asymmetrically deuterated sialyl *N*-glycans

The diacetyl strategy was applied to synthesize two asymmetrically deuterated sialyl *N*-glycans, **55** and **56**. Using deuterium-labeled *N*-glycan **55**, it was revealed that the neuraminidase derived from H1N1 preferentially cleaved sialic acids on α1,3-branched chains over α1,6-branched chains.

**FIGURE 13:**
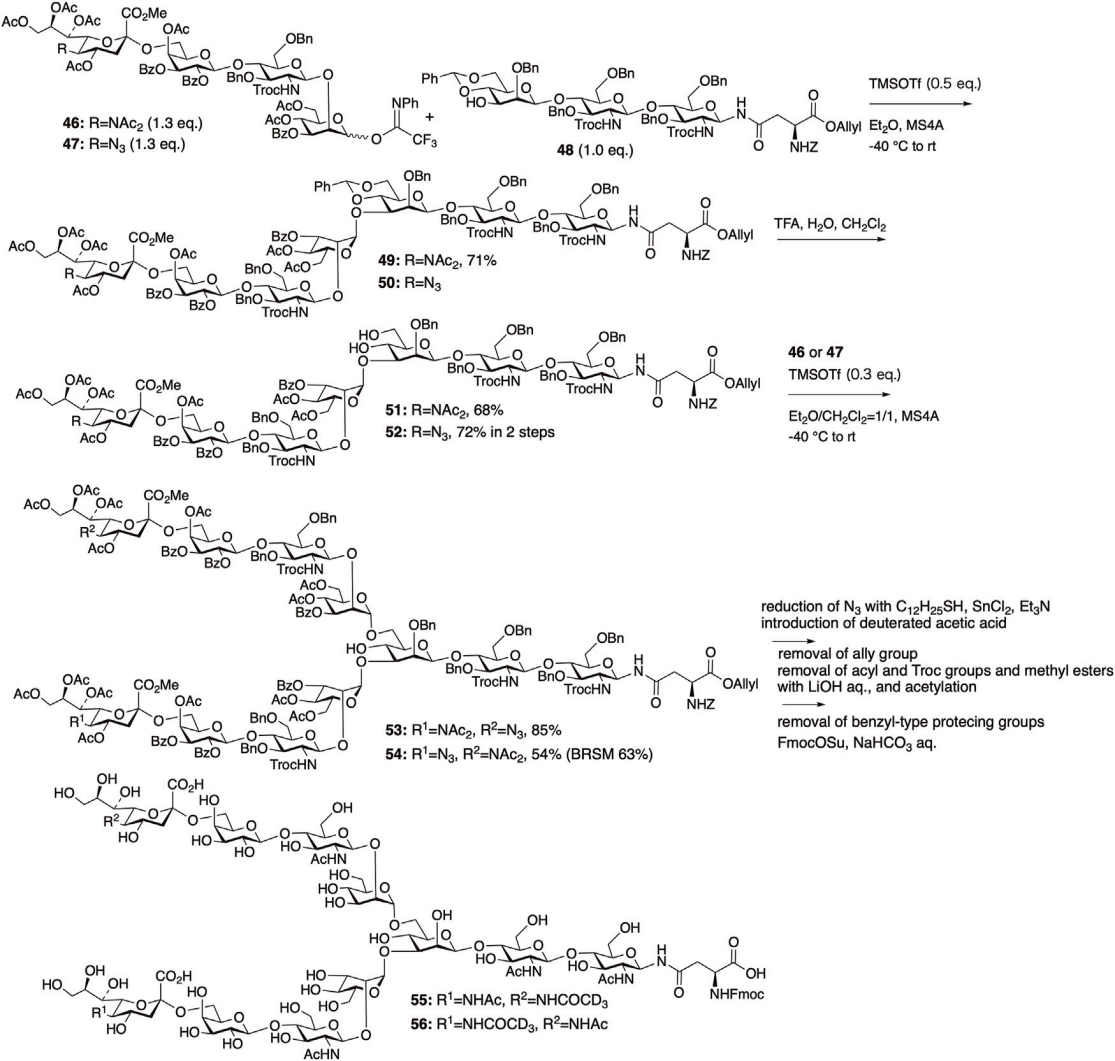
Synthesis of two biantennary *N*-glycans with asymmetrically deuterium-labeled sialic acid.

Deuterated *N*-glycans **55** and **56** were synthesized by glycosylation first at the 3-position of branching mannose in trisaccharide **48** with sialyl tetrasaccharides **46** or **47**, followed by glycosylation at the 6-position of branching mannose (Figure 13) (Shirakawa et al., 2021). We employed the remote participation method previously described by Kim et al. for mannosylation, in which acyl protection of the mannosyl donors at the *O*-3 and *O*-6 positions enhances α-selectivity (Baek et al., 2009).

In fact, the glycosylation between **46** and **48** proceeded smoothly with a stoichiometric amount of TMSOTf in Et_2_O to afford the desired heptasaccharide **49** in 71% yield with perfect α-selectivity. Glycosylation of the azide-containing sialyl tetrasaccharide **47** with **48**, followed by the deprotection of benzylidene in **50**, afforded **52** in good yield.

Because of the low solubility of sialyl glycan **51** obtained by the deprotection of **49** in Et_2_O, the [7 + 4] glycosylation between **51** and **47** was carried out in a mixed solvent system of Et_2_O/CH_2_Cl_2_ = 1/1, yielding the desired undecasaccharide **53** in 85% yield with perfect α-selectivity. Glycosylation between **52** and **46** under similar conditions afforded **54** in 54% yield (BRSM: 63%). Thus, we successfully constructed two asymmetric disialyl undecasaccharides, **53** and **54**, with one sialyl unit having an azide group available for deuterium labeling. The target deuterated *N*-glycans, **55** and **56**, were obtained by introducing a deuterated acetyl group, followed by global deprotection. Deuterium-hydrogen exchange was observed during the alkaline treatment for the removal of acyl and Troc groups, resulting in a decrease in the deuterium ratio of **55** to 42% and that of **56** to 63%, respectively.

#### 4.2.3 Synthesis of tetraantennary sialyl *N*-glycan

**FIGURE 14:**
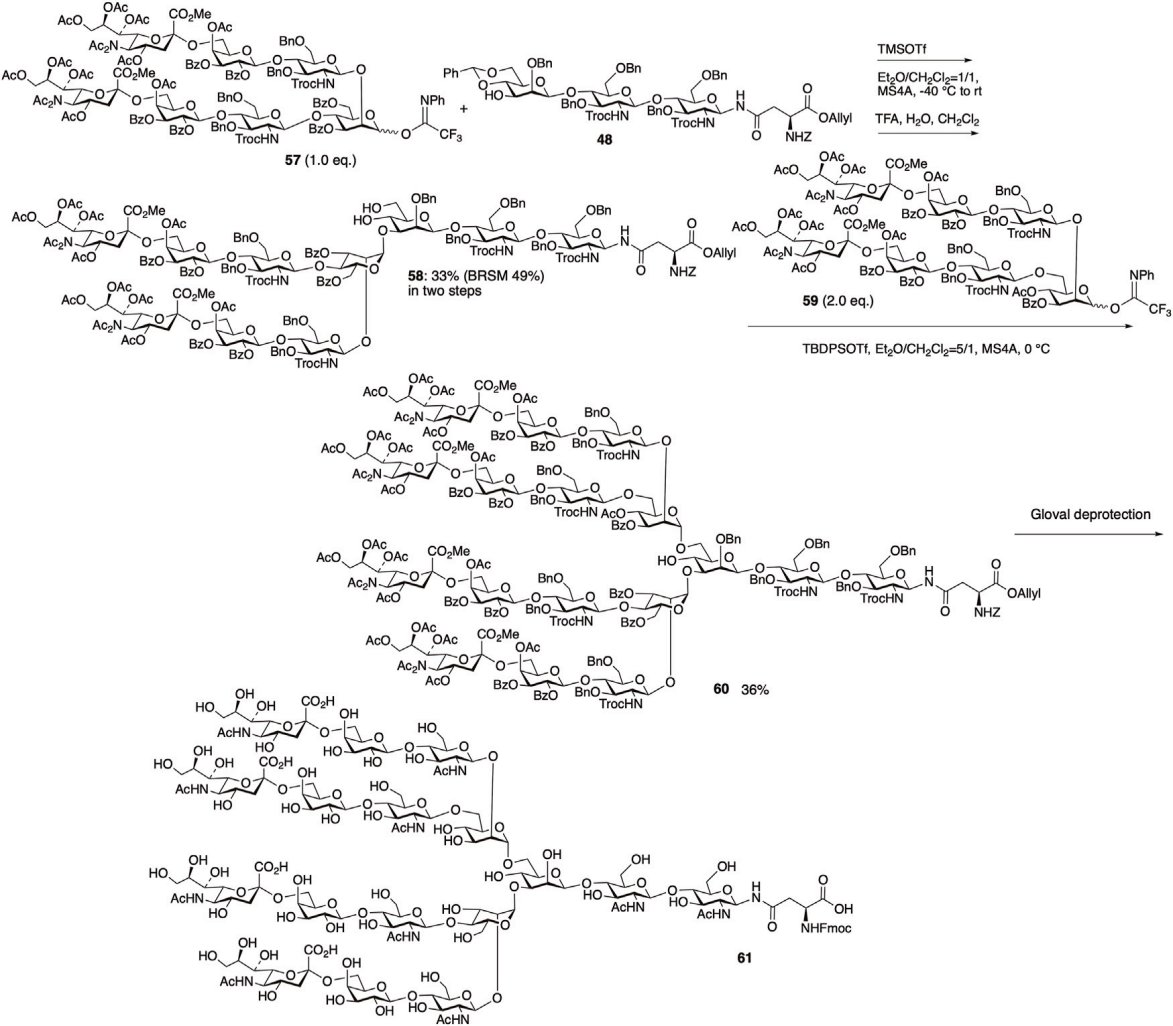
Synthesis of tetraantennary sialyl *N*-glycan.

Among the naturally occurring *N*-glycans, tetrasialylated *N*-glycan **61** is essential for evaluating the impact of multivalency and steric hindrance associated with the multiantennary structure. Fully sialylated tetraantennary *N*-glycan **61** was synthesized in a manner similar to those of **55** and **56** (Figure 14) (Shirakawa et al., 2021). The glycosylation between trisaccharide **48** and heptasaccharide donor **57** in the mixed solvent of Et_2_O/CH_2_Cl_2_ = 1/1 proceeded with complete α-selectivity, followed by cleavage of the benzylidene group, yielding decasaccharide **58** in 33% yield (BRSM: 49%) in two steps. The selection of the Lewis acid, solvent, and temperature played a crucial role in the subsequent glycosidation between decasaccharide **58** and heptasaccharide donor **59**. The glycosylation of **58** and **59** was achieved using TBDPSOTf at 0°C in a mixed solvent with a high ether ratio (Et_2_O/CH_2_Cl_2_ = 5/1), resulting in the formation of compound **60** with 36% yield. Following the deprotection of **60** and Fmoc introduction under conditions similar to the synthesis of **55** and **56**, we obtained fully sialylated tetraantennary *N*-glycan **61**.

In conclusion, the diacetyl strategy was instrumental in overcoming the challenges in sialyl glycan synthesis, allowing for the successful chemical synthesis of the asymmetrically deuterium-labeled sialyl *N*-glycans and the tetraantennary sialyl *N*-glycan.

## 5 Discussions

As mentioned above, the presence of NHAc groups in either glycosyl donors or acceptors significantly decreased the reaction rate of glycosylation. These phenomena are attributed to the decrease in the molecular motion of the substrates owing to intermolecular hydrogen bonding. Kononov et al. demonstrated that various protected saccharides form cluster structures termed supramers in solution, through intermolecular interactions mediated by hydrogen bonds and/or other non-covalent interactions between saccharides, and the formation of supramers significantly affects glycosylation reactivity. We observed that the effective volume of GlcNAc **17** was 1.2 times larger than that of *N,N*-diacetylated glucosamine (GlcN(Ac)_2_) **18** by DOSY experiments, indicating the formation of a supramer in the reaction solution through intermolecular hydrogen bonds. In the glycosylation of glycosyl donor **31** and glycosyl acceptor **32**, both of which have an NHAc group, the yield of sialic acid-containing tetrasaccharide **33** was 0% (Figure 8). Glycosylation reactions with either the donor or acceptor possessing an NHAc group afforded glycosylation products, despite the low yield. The notable difference in yields indicated an exceedingly low probability of collision between glycosyl donor **31** and glycosyl acceptor **32**. This outcome is suggested by the formation of supramers in both donor **31** and acceptor **32**, in addition to the decreased substrate mobility caused by intermolecular hydrogen bonds.

Myachin and Kononov reviewed the influence of reactor shape and flow rate on the efficiency and selectivity of reactions based on the formation of supramers and their dynamic behavior (Myachin and Kononov, 2023a; Myachin and Kononov, 2023b). In the Kdo glycosylation mentioned above, despite the absence of amide groups in the substrates, the efficiency of the glycosylation reaction increased when using a microflow reactor (Figure 1). This could be interpreted as microflow mixing disrupting the supramer structures in the solution to promote glycosylation.

In contrast, imide protection is used in the diacetyl strategy to prevent the formation of cluster structures via intermolecular hydrogen bonding, thus avoiding a decrease in reactivity. In our *N*-glycan synthesis, the Asn residue was bound to the reducing-end sugar. The amide group of the Asn side chain did not significantly affect synthetic efficiency. In the synthesis of the tetraantennary sialyl *N*-glycan, solubility decreased as the molecular size of the sialyl fragments increased. Consequently, glycosylation yields with such fragments were low. To improve the solubility of such fragments, it is necessary to change synthetic strategies, such as the selection of protective groups. Research focusing on solution structures in glycan synthesis is still in its early stages, and further advancements are anticipated in the future.
